# SFTSV utilizes AXL/GAS6 for entry via PI3K-PLC-dependent macropinocytosis activated by AXL-kinase

**DOI:** 10.1128/jvi.00221-25

**Published:** 2025-08-25

**Authors:** Zecheng Jin, Shuhei Taguwa, Junki Hirano, Kentaro Uemura, Chikako Ono, Akatsuki Saito, Tamaki Okabayashi, Yusuke Maeda, Taroh Kinoshita, Yoshiharu Matsuura

**Affiliations:** 1 Center for Infectious Disease Education and Research (CiDER), Osaka University 643214 , Osaka, Japan; 2 Research Institute for Microbial Diseases (RIMD), Osaka University, Osaka, Japan; 3 Center for Advanced Modalities and DDS (CAMaD), Osaka University, Osaka, Japan; 4 Department of Veterinary Science, Faculty of Agriculture, University of Miyazaki 12952, Miyazaki, Japan; Lerner Research Institute, Cleveland Clinic, Cleveland, Ohio, USA

**Keywords:** SFTSV, CRISPR activation screening, AXL, GAS6, auto-phosphorylation, PI3K, PLC, macropinocytosis

## Abstract

Severe fever with thrombocytopenia syndrome (SFTS) is a significant public health concern caused by SFTS virus (SFTSV), a tick-borne RNA virus. Previous studies have identified DC-SIGN and related C-type lectins as receptors of SFTSV infection in specific cell populations. Our genome-wide CRISPR activation screening identified AXL, a receptor tyrosine kinase, as a novel entry receptor for SFTSV. We found that AXL-mediated SFTSV infection utilizes the bridging action of growth arrest-specific protein 6 between AXL and phosphatidylserine on virus particles. This interaction induces autophosphorylation of tyrosine residues in the intracellular domain of AXL, recruiting phosphatidylinositol-3 kinase (PI3K) and phospholipase C (PLC) and establishing a macropinocytotic pathway of SFTSV entry. The AXL-PI3K-PLC-dependent entry pathway was observed in diverse cell types, including human umbilical vein endothelial cells (HUVEC), offering deep insights into the lifecycle of SFTSV and offering AXL as a novel therapeutic target against SFTS.

**IMPORTANCE:**

Understanding the molecular mechanisms of viral entry is critical for developing targeted antiviral therapies since there is no effective vaccine or antiviral drug against severe fever with thrombocytopenia syndrome (SFTS). This study uncovered AXL as a potential entry receptor for SFTS virus (SFTSV) via PI3K/PLC-dependent macropinocytosis pathway distinct from previously reported viral entry mechanism. The inhibition of these cellular enzymes resulted in the suppression of SFTSV infection in the AXL-expressing cell lines and HUVEC. Our research sheds light on the intricate molecular mechanisms underlying these interactions by utilizing mutants of AXL and represents a promising target for the development of innovative therapeutics against SFTS.

## INTRODUCTION

Severe fever with thrombocytopenia syndrome (SFTS), an emerging viral hemorrhagic fever, is a tick-borne infectious disease caused by the SFTS virus (SFTSV). Since SFTS was first observed in central China in 2009 (1), SFTSV case reports have increased and expanded in East Asia (2, 3). The typical clinical manifestations of SFTS are diverse, ranging from fever and gastrointestinal symptoms (3, 4) to more severe complications such as bone marrow examination revealing macrophage hemophagocytosis (hemophagocytic syndrome) (3) in severe cases. The fatality rate is alarmingly high, varying from 6.3% to 30% (3, 4).

SFTSV, classified as the family *Phenuiviridae*, order *Bunyavirales*, is a single-stranded, negative-sense RNA virus. SFTSV genome consists of three RNA segments (L, M, and S), each encoding RNA-dependent RNA polymerase, structural membrane glycoproteins (Gn and Gc), and nonstructural proteins (NP and NSs), respectively (4). Gn and Gc are envelope glycoproteins playing pivotal roles in virus entry and fusion to host cell membranes.

Three C-type lectin proteins, DC-SIGN (dendritic cell-specific intercellular adhesion molecule-3-grabbing non-integrin), DC-SIGNR (dendritic cell-specific intercellular adhesion molecule-3-grabbing non-integrin related, also known as L-SIGN, or liver/lymph node-specific intercellular adhesion molecule-3-grabbing non-integrin), and LSECtin (liver and lymph node sinusoidal endothelial cell C-type lectin), have been reported as host receptors for SFTSV infection (5–7). DC-SIGN binding depends on N-glycans on Gn and/or Gc (5). Recently, it was reported that in fatal SFTS patients, SFTSV was found mostly in macrophages and plasmablasts in the secondary lymphoid organs such as spleen and lymph nodes (LNs) (8). Thus, B cells/plasmablasts and macrophages were the major targets of SFTSV infection. Nevertheless, DC-SIGN was not detected in infected cells in LNs obtained from deceased individuals, and the plasmablasts in peripheral blood mononuclear cells isolated from healthy donors were less sensitive to SFTSV infection (8, 9). These facts suggest that host factors other than C-type lectins play roles in the SFTSV infection.

Here, we identified AXL as a receptor for SFTSV infection through comprehensive screening of a genome-wide clustered regularly interspaced short palindromic repeat (CRISPR)-activated (CRISPRa) library. AXL is a member of the TAM (Tyro3, AXL, MERTK) family of receptor tyrosine kinases (10), playing a pivotal role in regulating the phagocytic clearance of apoptotic cells and innate immune responses. This process involves the recognition of phosphatidylserine (PS) on the surface of apoptotic cells by the N-terminal γ-carboxyglutamate-rich (Gla) domain of growth arrest-specific protein 6 (GAS6) or protein S (PROS1) (11–13), followed by the binding of the C-terminal laminin-G like (LG1/2) domains of GAS6 to the immunoglobulin-like (Ig) domains of AXL (10, 11, 14, 15). This interaction leads to the autophosphorylation and activation of AXL, ultimately resulting in the formation of AXL-mediated signaling complexes (16). Recent studies have revealed the presence of PS on the envelopes of various viruses, including Lassa, Ebola, dengue, and Zika viruses, and demonstrated AXL’s involvement in these infections (17–25). Despite these advancements, the molecular basis of these infectious processes, particularly the functional significance of individual tyrosine residues of AXL in viral infection, remains inadequately explored.

Our study significantly advances understanding by delineating the essential roles of the 779th, 821st, and 886th tyrosine residues in AXL for SFTSV infection, leveraging a macropinocytosis pathway. This discovery sheds light on the intricacies of viral entry and underscores the potential of targeting AXL for therapeutic intervention in SFTSV and possibly other viral infections. These findings offer unprecedented insights into the molecular dynamics of AXL-mediated viral entry, opening new avenues for research and treatment strategies against SFTSV and similar pathogens.

## RESULTS

### Genetic screening for cellular proteins involved in SFTSV entry

**Fig 1:**
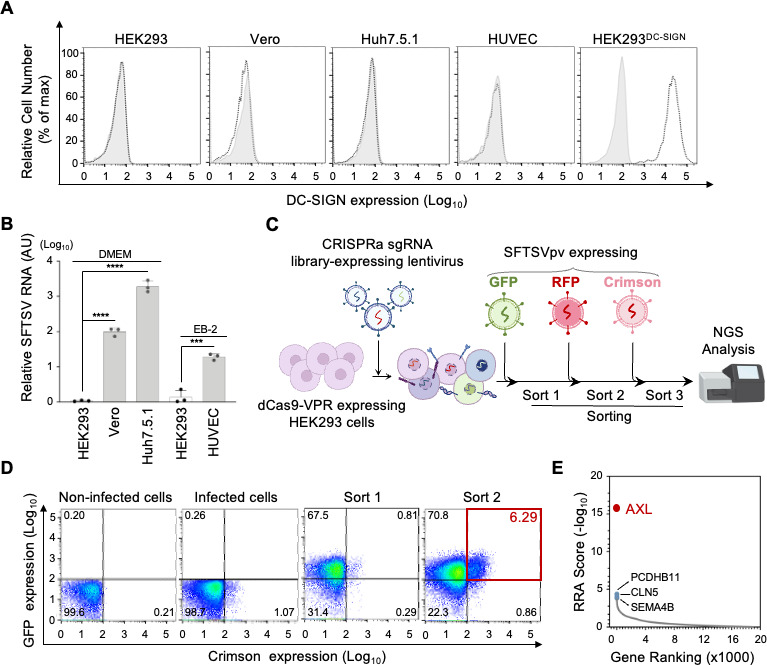
Genetic screening for cellular proteins involved in SFTSV entry. (**A**) Cell surface expression of DC-SIGN in HEK293, Vero, Huh7.5.1, HUVEC, and HEK293^DC-SIGN^ cells. HEK293^DC-SIGN^: HEK293 cells expressing DC-SIGN are used as positive control. (**B**) SFTSV infection (MOI: 0.01) in various cells. Total cellular RNA was extracted at 24 hpi, and relative SFTSV RNA levels compared to those of HEK293 cells were measured using RT-qPCR. Data shown are mean + SD of three independent experiments. Significance in panel **E** was determined by *t*-test; NS, not significant; ***P < 0.001; ****P < 0.0001. (**C**) Scheme depicting genome-wide CRISPR activation screening for potential receptors involved in SFTSVpv infection. In brief, reporter cell populations were generated by infecting HEK293 cells stably integrated with the doxycycline-inducible dCas9-VPR gene with a CRISPRa-v2 lentiviral pooled library. Among these reporter cell populations, cells more susceptible to SFTSVpv bearing fluorescent protein reporter genes were sorted based on reporter protein expression upon infection. Unsorted cells infected with SFTSVpv were used as control cell populations. After three rounds of sorting, genomic DNAs were extracted from sorted and unsorted cells, and the integrated sgRNAs were analyzed by deep sequencing and MAGeCK software. (**D**) Confirmation of enrichment of cells susceptible to SFTSVpv after two rounds of sorting. Cells before sorting and those after first and second sortings were infected with SFTSVpv harboring crimson reporter gene at an MOI of 0.01, and expression of crimson was analyzed using flow cytometry at 72 hpi. (**E**) *P*-values were calculated from the three sorting rounds and unsorted groups by a negative binomial model using a modified distribution of RRA algorithm named alpha-RRA. Of the top 30 candidates, several membrane protein genes are shown.

We first created an interferon (IFN)-alpha/beta receptor alpha chain (IFNAR1)-deficient human embryonic kidney 293 (HEK293) cell line (clone 14) via CRISPR-Cas9 to eliminate the confounding effects of type I IFN signaling on viral infection and propagation (Fig. S1A and B). This IFNAR1-knockout cell line, henceforth referred to as HEK293 cells, was used in subsequent experiments. In this study, we observed that Huh7.5.1 cells, human umbilical vein endothelial cells (HUVECs), and Vero cells were susceptible to SFTSV infection without the reported receptor DC-SIGN. This suggests that alternative cellular receptor(s) for SFTSV may exist (Fig. 1A and B). To identify potential unknown receptors, we utilized SFTSV-resistant HEK293 cells, lacking DC-SIGN expression and exhibiting resistance to SFTSV infection (Fig. 1A and B), as a genetic screening platform (Fig. 1C). We employed a pseudotype retrovirus system, incorporating SFTSV glycoproteins and fluorescent protein reporters (green, red, or crimson) to track SFTSV receptor expression. HEK293 cells were engineered to express inducible deactivated Cas9 (dCas9)-VPR (VP64, p65, and Rta) and subjected to a human genome-wide CRISPR sgRNA library, followed by a 2-week puromycin selection to enrich transduced cells. Before pseudovirus exposure, gene expression was activated with doxycycline (Dox). Control populations were maintained without sorting. Green fluorescent protein (GFP)-positive cells were sorted at 72 h post-infection (hpi) following infection with GFP-expressing SFTSV pseudovirus (SFTSVpv) at a multiplicity of infection (MOI) of 0.01 (Fig. 1C, Sort 1). A second sort (Sort 2) was conducted following additional cell propagation for cells expressing red fluorescent protein (RFP) post-infection with RFP-expressing SFTSVpv. Flow cytometry revealed a 6.29% increase in cells responsive to SFTSVpv expressing crimson (Fig. 1D). Subsequent RNAseq analysis of enriched sgRNAs from the third round of sorting (green, red, and crimson-positive cells) identified four membrane protein genes (*AXL*, *CLN5*, *PCDHB11*, and *SEMA4B*) that were associated with the top 30 candidates (Fig. 1E). Notably, *AXL* exhibited the most significant enrichment, reflected by the lowest robust rank aggregation (RRA) score and a false discovery rate of <0.005 for *AXL* (Table S1).

### AXL participates in SFTSV entry and synergistically enhances viral infection with DC-SIGN

**Fig 2:**
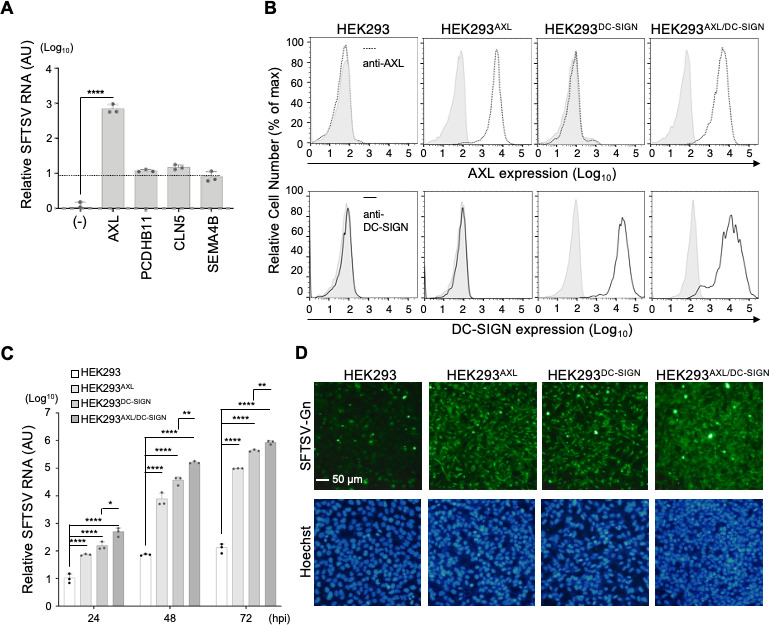
AXL participates in SFTSV entry and synergistically enhances virus infection with DC-SIGN. (**A**) Susceptibility of HEK293 cells expressing candidate genes selected in Fig. 1E. HEK293 cells expressing these candidate genes were infected with SFTSV (MOI = 0.01) at 37°C for 2 h, and amounts of SFTSV RNA relative to those of parent HEK293 cells were determined using RT-qPCR at 72 hpi. Data shown are mean ± SD of three independent experiments. (**B**) HEK293 cell lines stably expressing either AXL (HEK293^AXL^) or DC-SIGN (HEK293^DC-SIGN^) and both proteins (HEK293^AXL/DC-SIGN^) were established, and surface expression of AXL and DC-SIGN was determined using flow cytometry. (**C**) Expression of AXL and DC-SIGN independently and synergistically enhanced SFTSV infection. Cells infected with SFTSV at an MOI of 0.01 for 2 h and total cellular RNAs were extracted at 24, 48, and 72 hpi. Amounts of SFTSV RNA relative to those in parental HEK293 cells were determined using RT-qPCR. Data shown are mean ± SD of three independent experiments. (**D**) Cells described in panel B were infected with SFTSV at an MOI of 50, and expression of SFTSV Gn protein (green) was determined by fluorescent microscopy at 24 hpi. Nuclei were stained by Hoechst 33342 (blue). Scale bar, 50 µm. Significance in panels **A** and **C** was determined by *t*-test; NS, not significant; *P < 0.05; **P < 0.01; ***P < 0.001; ****P < 0.0001.

To examine the contribution of identified host proteins to SFTSV entry, we transduced HEK293 cells with retroviruses carrying each candidate gene, followed by SFTSV infection. RT-qPCR determined SFTSV RNA levels upon infection at 72 hpi (Fig. 2A). Cells overexpressing AXL significantly increased viral RNA production—approximately 700-fold over parental HEK293 cells and 70-fold over cells expressing CLN5, PCDHB11, or SEMA4B. Therefore, we focused on AXL for further study. To examine the synergistic effect of AXL on the function of the known entry receptor DC-SIGN, we established stable HEK293 cell lines expressing either AXL (HEK293^AXL^) or DC-SIGN (HEK293^DC-SIGN^) (Fig. 1A; Fig. S1C) and both proteins (HEK293^AXL/DC-SIGN^). We verified their surface expression using flow cytometry (Fig. 2B). Next, these cells were infected with SFTSV at an MOI of 0.01, and propagation of viral RNA was determined using RT-qPCR at 24, 48, and 72 h post-infection. SFTSV RNA expression in cells increased with time during infection in the descending order of HEK293^AXL/DC-SIGN^, HEK293^DC-SIGN^, HEK293^AXL^, and parental cells (Fig. 2C). Additionally, immunofluorescence microscopy assessing the expression of SFTSV Gn protein at 24 hpi corroborated these findings (Fig. 2D). Our data suggest that AXL and DC-SIGN collectively augment SFTSV infection, potentially through distinct downstream signaling pathways.

### AXL mediates SFTSV entry by binding to PS in the viral envelope via GAS6

**Fig 3:**
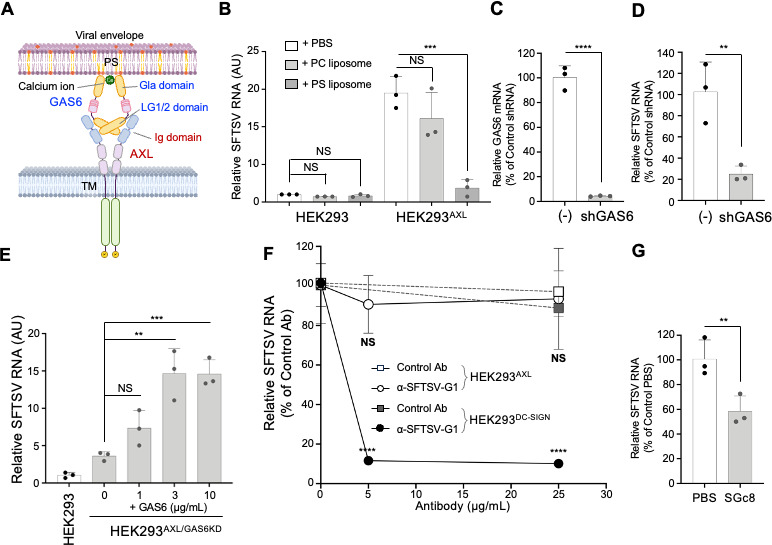
AXL mediates SFTSV entry by binding to PS in the viral envelope via GAS6. (**A**) Schematic showing dimerization of AXL mediated by Ca-dependent binding of GAS6 to PS in the viral envelope. GAS6 recognizes PS by the Gla domain and interacts with AXL by the LG1/2 domain. (**B**) PS-liposomes competitively inhibit AXL-mediated SFTSV infection. HEK293 and HEK293^AXL^ cells were preincubated with either PC-liposome, PS-liposome, or PBS on ice for 15 min and were infected with SFTSV at an MOI of 0.1 at 37°C for 2 h. Total cellular RNAs were extracted at 6 hpi, and the amounts of SFTSV RNA relative to those of HEK293 cells incubated in PBS were determined using RT-qPCR. Data shown are mean ± SD of three independent experiments. (C–E) GAS6 is a critical factor in AXL-mediated SFTSV infection. GAS6 expression in HEK293^AXL^ cells was reduced by lentivirus-based shRNAi (HEK293^AXL/GAS6KD^), and GAS6 transcript level was compared with that of control HEK293^AXL/vector^ cells (−) that were infected with empty lentivirus-based shRNAi vector (**C**). HEK293^AXL/vector^ cells (−) and HEK293^AXL/GAS6KD^ cells were cultured in 2% FBS-DMEM for 12 h and then in serum-free medium for 1 h. These cells were infected with SFTSV (MOI = 0.1) at 37°C for 2 h. Total cellular RNAs were extracted at 24 hpi, and the amounts of SFTSV RNA were determined (**D**). HEK293^AXL/GAS6KD^ cells in serum-free medium were supplemented with GAS6 (1, 3, and 10 mg/mL) and infected with SFTSV at an MOI of 0.1 at 37°C for 2 h. Total cellular RNAs were extracted at 24 hpi, and the amounts of SFTSV RNA relative to those in HEK293 cells without GAS6 were determined using RT-qPCR (**E**). Data shown are mean ± SD of three independent experiments. (**F**) Inhibition of SFTSV infection of HEK293^DC-SIGN^ but not of HEK293^AXL^ by anti-SFTSV-G1 antibody recognizing an envelope glycoprotein Gn. SFTSV (MOI = 0.1) was preincubated with SFTSV-G1 antibody or control IgG at 4°C for 1 h, and HEK293^AXL^ and HEK293^DC-SIGN^ cells were challenged with these pre-treated SFTSVs at 37°C for 2 h. Total cellular RNAs were extracted at 24 hpi, and the amounts of SFTSV RNA relative to those not treated with antibody were determined using RT-qPCR. Data shown are mean ± SD of three independent experiments. (**G**) Inhibition of SFTSV infection of HEK293^AXL^ by SFTSV-Gc target peptide SGc8. SFTSV (MOI = 0.1) premixed with buffer or 50 µM SGc8 at 4°C was added to HEK293^AXL^ and incubated at 37°C for 2 h. The total cellular RNA was extracted at 24 hpi, and relative SFTSV RNA levels were determined using RT-qPCR. Data shown are mean ± SD of three independent experiments. Significance in panels **B** to **G** was determined by *t*-test; NS, not significant; *P < 0.05; **P < 0.01; ***P < 0.001; ****P < 0.0001.

To discern whether AXL-mediated SFTSV entry parallels the entry mechanisms of other viruses, such as the Lassa virus, Ebola virus, dengue virus, and Zika virus, involving PS on the viral envelope and GAS6, we assessed the roles of these components. GAS6 recognizes PS by the Gla domain and interacts with AXL by the LG1/2 domain (Fig. 3A). Pre-treatment with PS-liposomes diminished SFTSV infection in HEK293^AXL^ cells by roughly 80%, unlike phosphatidylcholine (PC)-liposomes, indicating the specificity of the PS-AXL interaction (Fig. 3B). To address the potential confounding effects of GAS6 present in serum and produced by cells, we established GAS6-knockdown HEK293^AXL^ cells (HEK293^AXL/GAS6KD^) (Fig. 3C). Replication of SFTSV RNA in HEK293^AXL/GAS6KD^ was impaired compared to the control cells (Fig. 3D). In HEK293^AXL/GAS6KD^ cells, the addition of recombinant GAS6 restored SFTSV infection in a dose-dependent manner, highlighting an essential role of GAS6 in AXL-mediated viral entry (Fig. 3E). Further probing the entry mechanisms, we examined an anti-SFTSV-G1 neutralizing antibody (26). The antibody inhibited infection in DC-SIGN-expressing cells but not in AXL-expressing cells, indicating a unique SFTSV Gn-independent, AXL-mediated entry pathway (Fig. 3F). The Gc-derived peptide SGc8 (27) also reduced SFTSV infection in AXL-expressing cells, suggesting Gc-mediated fusion in AXL-dependent infection (Fig. 3G). Our data support a model where AXL-mediated SFTSV entry necessitates the bridging action of GAS6 between AXL and PS on the viral envelope.

### Kinase activity and autophosphorylation of tyrosine residues in an intracellular domain of AXL are critical for SFTSV entry

**Fig 4:**
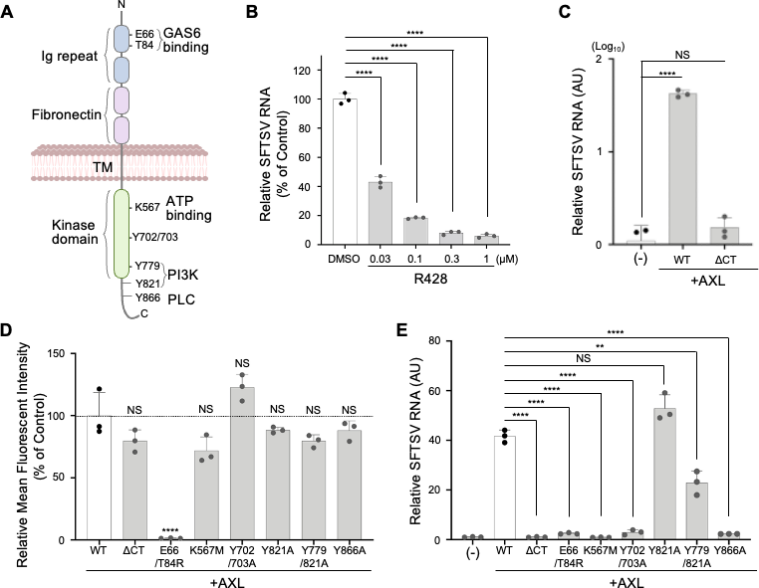
Kinase activity and autophosphorylation of tyrosine residues in an intracellular domain of AXL are critical for SFTSV entry. (**A**) Schematic diagram of AXL structure. (**B**) R428 suppressed SFTSV infection in a dose-dependent manner. HEK293^AXL^ cells were preincubated for 1 h with the indicated concentration of R428 (0.03–1 µM) or dimethyl sulfoxide (DMSO) and then infected with SFTSV (MOI = 0.1) in the presence of R428 for another 2 h at 37°C. Total cellular RNAs were extracted at 24 hpi, and SFTSV RNA level relative to that of HEK293^AXL^ cells treated with DMSO was determined using RT-qPCR. Data shown are mean ± SD of three independent experiments. (**C**) AXL C-terminal region including the kinase domain is essential for SFTSV infection. HEK293 (−), HEK293^AXL^, and HEK293^AXL-∆CT^ cells were infected with SFTSV (MOI = 0.1) at 37°C for 2 h. Total cellular RNAs were extracted at 36 hpi, and SFTSV RNA level relative to that in HEK293 cells was determined using RT-qPCR. Data shown are mean ± SD of three independent experiments. (**D and E**) Characterization of AXL mutants for GAS6 binding and SFTSV infection. HEK293 cells expressing various AXL mutants were incubated with 1 µg/mL of GAS6-Flag for 1 h at 4°C, and binding of GAS6-Flag was determined by a flow cytometer with an anti-Flag antibody and displayed as the mean fluorescence intensity ± SD from three independent experiments (**D**). HEK293 cells expressing various AXL mutants were infected with SFTSV (MOI = 0.1) for 2 h at 37°C. Total cellular RNAs were extracted at 72 hpi, and SFTSV RNA level relative to that of HEK293 cells was determined using RT-qPCR (**E**). Data shown are mean ± SD of three independent experiments. Significance in panels **C** to **E** was determined by *t*-test; NS, not significant; **P < 0.01; ****P < 0.0001.

AXL consists of extracellular Ig and fibronectin domains, a transmembrane domain, and an intracellular kinase domain with multiple tyrosine phosphorylation sites (Fig. 4A). We explored the necessity of the kinase function of AXL in SFTSV entry using R428, an AXL kinase inhibitor, and an AXL mutant lacking the intracellular kinase domain (AXL-ΔCT). R428 treatment inhibited SFTSV infection dose-dependently (Fig. 4B; Fig. S2A), and AXL-ΔCT expressing HEK293 cells (HEK293^AXL-ΔCT^) showed significantly lower infection rates compared to full-length AXL-expressing cells (HEK293^AXL^) (Fig. 4C; Fig. S2B). This reduced susceptibility to SFTSV occurred despite similar levels of AXL expression on the cell surface and GAS6 binding capacities (Fig. S2C and D), suggesting that the kinase activity of AXL participates in SFTSV infection.

To determine the roles of the intracellular tyrosine residues of AXL in SFTSV entry, we generated HEK293 cell lines stably expressing various AXL mutants. Autophosphorylation of tyrosine at positions 698, 702, 703, 779, 821, and 866 occurs upon PS-GAS6 activation of AXL (28). Notably, lysine-to-methionine substitution at position 567 (K567M) in the kinase domain yields a kinase-inactive AXL variant (29). Tyrosines 702 and 703, located in the activation loop, are crucial for kinase activation; their alanine substitution (Y702/703A) negates kinase function (28, 29). Tyrosine 821, a phosphorylation hotspot, interacts with several signaling molecules, including the phosphatidylinositol-3 kinase (PI3K) subunit p85, phospholipase C (PLC), and others (16, 29). The phosphorylation sites at 779 and 866 of AXL specifically associate with PI3K p85 and PLC, respectively (16, 29). Altering glutamic acid 66 and threonine 84 to arginine (E66/T84R) in the first Ig domain disrupts GAS6 binding (11). Among the cell lines expressing AXL mutants, only HEK293^AXL-E66/T84R^ abrogated binding activity to GAS6, leading to resistance to SFTSV infection, despite cell surface expression of AXL (Fig. 4D; Fig. S2C). Kinase-deficient mutants HEK293^AXL-ΔCT^, HEK293^AXL-K567M^, and HEK293^AXL-Y702/703A^ displayed negligible SFTSV infection (Fig. 4E). While the Y821A mutation in AXL did not affect SFTSV infection, consistent with the previous reports on Ebola virus (18, 25) and Lassa virus (21), the Y779/821A double mutation in AXL significantly reduced susceptibility to SFTSV, likely due to a loss of PI3K interaction. Interestingly, the AXL^Y866A^ mutation, which disrupts PLC binding, significantly impeded SFTSV infection (Fig. 4E). These results indicate that kinase activity and autophosphorylation of tyrosine residues in the intracellular domain of AXL are required for SFTSV infection.

### PI3K-, PLC-, and macropinocytosis- but not clathrin/dynamin-dependent pathways are involved in AXL-mediated SFTSV entry

**Fig 5:**
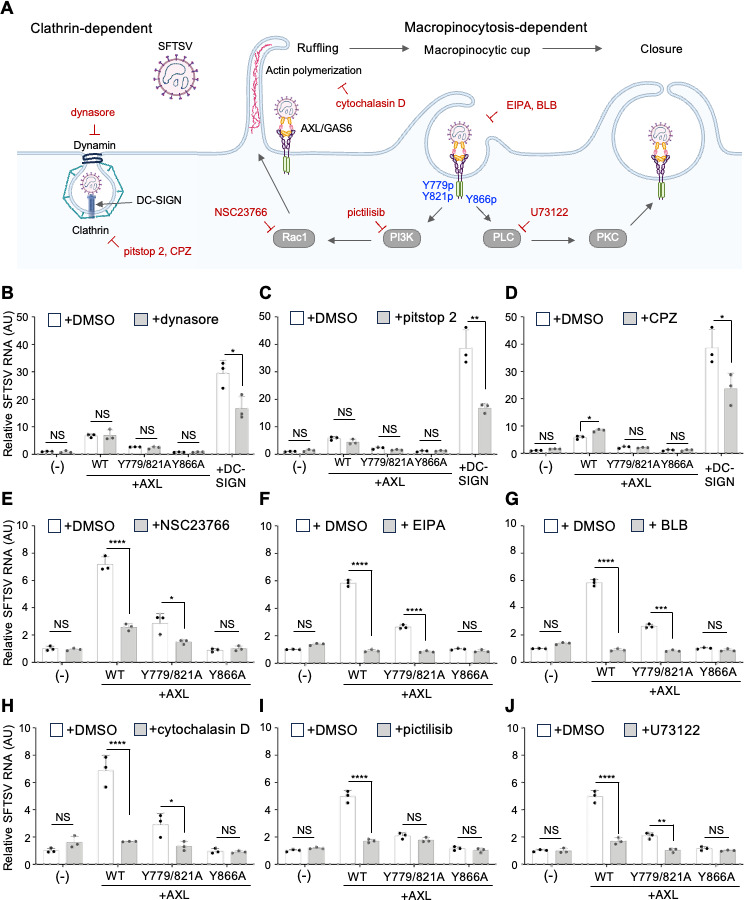
PI3K-, PLC-, and macropinocytosis- but not clathrin/dynamin-dependent pathways are involved in AXL-mediated SFTSV entry. (**A**) Inhibitors tested for AXL-dependent SFTSV infection. (B–J) HEK293 (−), HEK293^AXL^, HEK293^AXL-Y779/821A^, HEK293^AXL-Y866A^, and HEK293^DC-SIGN^ cells were preincubated with either 50 µM dynasore (**B**), 20 µM pitstop2 (**C**), 10 µM CPZ (**D**), 100 µM NSC23766 (**E**), 30 µM EIPA (**F**) or 40 µM BLB (**G**), 10 µM cytochalasin D (**H**), 5 µM pictilisib (**I**), 1 µM U73122 (**J**) for 1 h at 37°C and infected with SFTSV (MOI = 0.1) at 37°C in the presence of the inhibitors. After 2 h of incubation, the cells were washed three times, followed by incubation for another 6 h in the presence of the inhibitors, and SFTSV RNA level relative to that of HEK293 cells was determined using RT-qPCR. Data shown are mean ± SD of three independent experiments. Significance in panels **B** to **J** was determined by *t*-test; NS, not significant; *P < 0.05; **P < 0.01; ***P < 0.001; ****P < 0.0001.

To examine the involvement of the tyrosine residues in AXL in endocytosis and macropinocytosis, we investigated the effects of various inhibitors of endocytosis, micropinocytosis, PI3K, and PLC (Fig. 5A). Inhibitors of dynamin (dynasore) and clathrin-mediated endocytosis (pitstop 2 and chlorpromazine; CPZ) exhibited no significant effect on SFTSV infection in HEK293^AXL^ cells, although they reduced infection in HEK293^DC-SIGN^ cells (Fig. 5B through D), suggesting that AXL-mediated entry does not rely on clathrin/dynamin-dependent pathways. In contrast, treatment with inhibitors of macropinocytosis, including the Rac1 inhibitor NSC23766, the Na+/H+ exchanger inhibitor 5‐(N‐ethyl‐N‐isopropyl) amiloride (EIPA), the non-muscle myosin II inhibitor blebbistatin (BLB), the actin polymerization inhibitor cytochalasin D, significantly curtailed SFTSV infection in HEK293^AXL^ and HEK293^AXL-Y799/821A^ cells but not in HEK293^AXL-Y866A^ (Fig. 5E through H). To further examine the effects of PI3K and PLC inhibitors on SFTSV entry, we treated HEK293 cells stably expressing various AXL mutants. Treatment with pictilisib, a PI3K inhibitor, markedly reduced SFTSV infection of HEK293^AXL^ but not that of HEK293^AXL-Y779/821A^ and HEK293^AXL-Y866A^, suggesting that AXL-Y779/821A mutation suppresses SFTSV infection through an impaired recruitment and/or activation of PI3K (Fig. 5I). Treatment with U73122, a PLC inhibitor, significantly reduced SFTSV infection of HEK293^AXL^ and HEK293^AXL-Y779/821A^ but not of HEK293^AXL-Y866A^, indicating that AXL-Y866A mutation inhibits SFTSV infection due to loss of PLC binding (Fig. 5J). These data suggest that macropinocytosis participates in the AXL-mediated SFTSV entry.

PI3K inhibitor exhibited no effect on SFTSV infection in HEK293^AXL-Y866A^ (defective in PLC binding), while PLC inhibitor suppressed infection in HEK293^AXL-Y779/821A^ (defective in PI3K binding), suggesting that PLC is functionally downstream of PI3K. Notably, treatment with either EIPA or BLB exhibited no synergistic effect on SFTSV infection to HEK293^AXL-Y866A^, suggesting a crucial role of Y866 phosphorylation in the PLC-dependent macropinocytotic pathway of SFTSV entry.

### SFTSV infection of HUVEC also utilizes AXL

**Fig 6:**
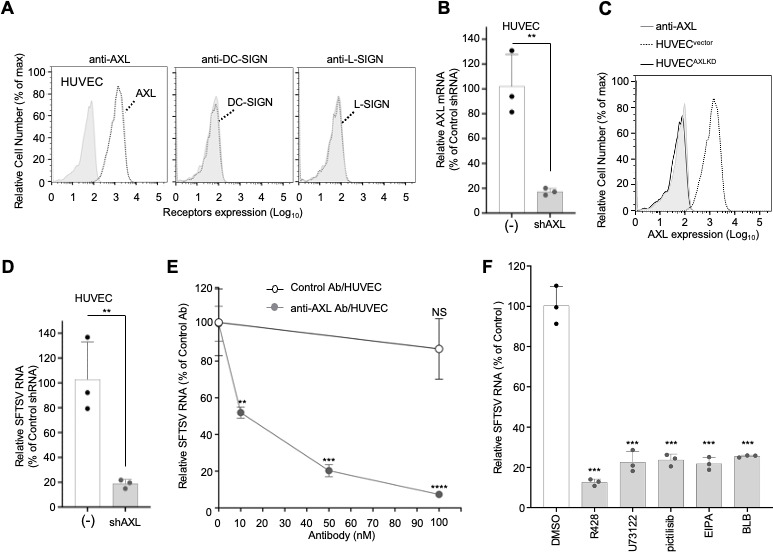
SFTSV infection to HUVEC also utilizes AXL. (**A**) Cell surface expression of AXL and lectin (DC-SIGN and L-SIGN) family members in HUVEC was analyzed using flow cytometry. (B–D) AXL expression is required for SFTSV infection in HUVECs. HUVECs were transduced by lentivirus encoding shRNAi targeting AXL (HUVEC^AXLKD^) or empty vector [HUVEC^vector^ (−)], and expression of AXL was determined using RT-qPCR (**B**) and flow cytometry (**C**). HUVEC^AXLKD^ and HUVEC^vector^ (−) were infected with SFTSV (MOI = 0.1) for 2 h at 37°C, and SFTSV RNA level relative to that in HUVEC^vector^ cells (−) was determined using RT-qPCR at 24 hpi (**D**). Data shown are mean ± SD of three independent experiments. (**E**) HUVECs preincubated with anti-AXL antibody or control IgG for 1 h at 4°C were challenged with SFTSV (MOI = 0.1) for 2 h at 37°C in the presence of antibodies. SFTSV RNA level relative to that of HUVEC without antibody was determined using RT-qPCR at 24 hpi. Data shown are mean ± SD of three independent experiments. (**F**) Effects of inhibitors on SFTSV infection in HUVECs. The same procedure was used as that in Fig. 5. Data shown are mean ± SD of three independent experiments. Significance in panels **C** to **F** was determined by *t*-test; NS, not significant; **P < 0.01; ***P < 0.001; ****P < 0.0001.

HUVEC, a widely used primary endothelial cell model for viral infection mechanism, exhibits susceptibility to SFTSV (Fig. 1B) and expresses AXL but lacks DC-SIGN/L-SIGN expression (Fig. 1A; Fig. 6A). We knocked down AXL in HUVECs using lentiviral shRNA to create HUVEC^AXLKD^, leading to a decrease in both mRNA and cell surface expression of AXL, as confirmed by RT-qPCR and flow cytometry (Fig. 6B and C). This AXL knockdown significantly attenuated SFTSV susceptibility in HUVEC^AXLKD^ compared to the control HUVEC^vector^ (Fig. 6D). To further validate the role of AXL in SFTSV infection, HUVECs were preincubated with anti-AXL antibody AF154, which blocks GAS6 interaction, and challenged with SFTSV. Treatment with the anti-AXL antibody neutralized infection in a dose-dependent manner, suggesting that SFTSV utilizes AXL for entry into HUVECs (Fig. 6E). Furthermore, SFTSV infection to HUVECs was significantly inhibited by the treatment with R428 (AXL kinase inhibitor), pictilisib (PI3K inhibitor), U73122 (PLC inhibitor), EIPA (macropinocytosis inhibitor), and BLB (myosin II inhibitor) (Fig. 6F) as seen in HEK293^AXL^ cells, suggesting that SFTSV utilizes AXL for entry to various types of cells through the PI3K- and PLC-dependent micropinocytosis.

## DISCUSSION

PS constitutes a primary phospholipid within cell membranes, typically confined to the inner leaflet of the lipid bilayer (30). During abnormal conditions, including cell death, scramblases facilitate its translocation from the inner to the outer leaflet, positioning it on the cell surface and acting as an “eat me” signal (31) crucial for phagocytic cell clearance. AXL plays a role in cellular signaling pathways influencing cell survival (32–34), proliferation (35–37), and immune responses (38, 39). Consequently, these receptors have been targeted for drug development, especially in the fields of cancer therapy (40–42) and immunotherapy (43).

It is widely recognized that AXL expression significantly influences viral proliferation by serving as a phagocytic receptor for apoptotic cells in a process known as viral apoptotic mimicry (44–46). However, the role of AXL kinase activity and its mechanism of entry exhibits variability across different viruses. Previously reported viruses such as the Lassa virus (21, 47, 48), Ebola virus (18, 25, 49), dengue virus (19, 24), and Zika virus (22–24, 50) have been documented to infect cells through AXL interaction indirectly via the formation of PS-GAS6-AXL complexes on the virus surface. Notably, for flaviviruses, it was reported that the AXL kinase activity was not involved in AXL-mediated viral entry but played a crucial role in post-entry events, especially in viral replication by inhibition of the IFN pathway (22, 31, 51). Recent research has revealed AXL’s function as an entry receptor for severe acute respiratory syndrome coronavirus 2 (SARS-CoV-2) in lung and bronchial epithelial cells (51). Intriguingly, the N-terminal domain of the SARS-CoV-2 spike protein exhibits relatively low affinity (dissociation constant (K_D_ = K_on_/K_off_), K_D_ = 882 nM) for direct binding with AXL (51). Additionally, AXL has been identified as a co-receptor for human parvovirus B19 (B19V), displaying a high affinity (K_D_ = 103 nM) for direct interaction with the B19V capsid protein VP1u (52). The extensive expression of AXL across various human tissues, as documented in the Human Protein Atlas database (https://www.proteinatlas.org), combined with its involvement in multiple stages from entry to immune evasion in the lifecycle of viruses like the Ebola virus (49) and flaviviruses (53), provides a solid foundation for considering its role in SFTSV infection. Given the broad tissue distribution of AXL, surpassing that of DC-SIGN and including key cell types such as fibroblasts and Langerhans cells (52) in the skin, the primary sites for vector-borne viral entry, AXL emerges as a primary receptor for SFTSV in the early stages of infection. This early infection phase, crucial for virus dissemination, remains underexplored. Therefore, focusing on AXL aligns with the established patterns of viral entry and immune evasion and highlights its attractiveness as a molecule for therapeutic targeting against SFTSV, offering a promising avenue for future research and potential treatment strategies.

The entry of flaviviruses through AXL is mediated by clathrin-dependent endocytosis (51), and the macropinocytosis pathway has not been reported. Conversely, in Lassa (48) and Ebola (49) viruses, the entry through AXL depends on AXL kinase activity and via macropinocytosis, which is also utilized in cancer cells for AXL-dependent invasion (16, 28, 53–56). Moreover, this highlights a potential research gap regarding the interaction between AXL kinase activity and IFN pathway in viral infections, aside from one report describing that treatment with R428 did not increase IFN-I response upon arenavirus infection (16). Importantly, we used IFNAR1 knockout HEK293 cells in all experiments, and the effect of IFN pathway can be disregarded. AXL mutants (ΔCT and K567M) and R428 treatment significantly reduced SFTSV proliferation, suggesting a potential disruption in viral entry. We examined the endocytosis mode of AXL-mediated SFTSV entry by using inhibitors such as CPZ and dynasore for clathrin/dynamin-dependent pathway and EIPA and BLB for micropinocytosis. We found that AXL-mediated SFTSV entry mainly relies on macropinocytosis. Multiple reports (5, 6) support that SFTSV likely used clathrin/dynamin-mediated endocytosis, although the receptor molecules were unassigned, suggesting that SFTSV internalizes into various cells depending on the presence of receptors.

Substitution of tyrosine residues in the kinase domain of AXL to alanine and treatment with endocytosis inhibitors revealed the relationship between the phosphorylation sites and viral endocytosis/proliferation. The Y821A single mutant exhibited no effect on the AXL-mediated infection, consistent with previous results using glycoproteins from Ebola and Lassa viruses (23, 24). However, dual mutation of Y779 and Y821, both tyrosine residues that participate in PI3K binding, moderately inhibited the infection. In contrast, the Y866A mutation abrogated SFTSV infection, and phosphorylation of Y886 participates in PLC binding (43, 57). Treatment of cells expressing wild-type and Y779/821A but not of Y866A mutant with PLC inhibitor U73122 reduced the susceptibility to SFTSV (Fig. 5J), indicating that phosphorylation of Y886 plays a pivotal role in SFTSV infection, and PLC was functionally downstream of PI3K. Notably, treatment with macropinocytosis inhibitors significantly reduced the AXL-mediated SFTSV infection but did not show any synergistic inhibitory effects on the Y866A mutation. These data support a mechanism that PLC recruited by the phosphorylated Y866 is activated by PI3K recruited by the phosphorylated Y779/821 and eventually initiates macropinocytosis. However, Y779- and Y821-independent AXL-mediated SFTSV infection was completely suppressed by the treatment with PI3K or PLC inhibitors. Crucially, this scenario aligns with the proposed macropinocytosis controlled by different classes of phosphoinositides involving PI3K, PLC (55, 58), and actin rearrangement (Fig. 5A) (59–62). Receptor tyrosine kinases recruit class I PI3K to the cell membrane via tyrosine phosphorylation, forming phosphatidylinositol 3,4,5-trisphosphate [PI(3,4,5)P_3_] clusters where macropinocytic cups are formed (63). PI(3,4,5)P_3_ is essential for PLC recruitment and activation. During closure, PLC partially intervenes, hydrolyzing phosphatidylinositol 4,5-bisphosphate [PI(4,5)P_2_] and/or PI(3,4,5)P_3_, producing diacylglycerol. Furthermore, PI(3,4,5)P_3_ is hydrolyzed into phosphatidylinositol (3,4)-bisphosphate [PI(3,4)P_2_] by a phosphatase SHIP2 (64, 65) in the macropinosome, converted to phosphatidylinositol 3-phosphate [PI(3)P] by inositol polyphosphate 4-phosphatase type II (66, 67), where Ras-related protein Rab5a is recruited. Genetic and pharmacological approaches both indicate the indispensable role of class I PI3K in completing macropinosome closure, hence substantially supporting the roles of PI3K and PLC, as highlighted in our results.

In summary, we found that AXL functions as a new receptor for SFTSV by its binding to the complex of GAS6 and PS, which was exposed on the surface of SFTSV particles, and that SFTSV is endocytosed through the macropinocytosis pathway, which requires kinase activity of AXL. Future studies are needed to elucidate the *in vivo* role of AXL in animal models and its relationship to other PS-binding proteins. R428, also known as bemcentinib (BGB324), has been undergoing clinical trials in the cancer field (68). Inhibitors for AXL-mediated SFTSV entry can be used as novel therapeutics for SFTS patients.

## MATERIALS AND METHODS

### Viruses

SFTSV (Miyazaki strain) (69) was amplified in Vero cells and stocked at −80°C in aliquots. The infectious titer was determined by plaque assay, as described below. All SFTSV infection and amplification experiments were performed in a biosafety level 3 (BSL3) containment facility. Virus stock was diluted for viral infection at desired concentrations in 2% fetal bovine serum (FBS)-Dulbecco’s modified Eagle medium (DMEM) or endothelial basal medium (EBM-2). Cells on collagen-coated 48-well plates (Iwaki Science Products Dept., Shizuoka, Japan) were incubated with the culture medium containing diluted virus stock solution at 37° for 2 h. After removal of the medium, the cells were washed three times with 2% FBS-DMEM or EBM-2 and further cultured in a fresh medium. At ~24–72 hpi, the cells were harvested for RNA extraction.

### Cells and culture

HEK293 and HEK293T cells (American Type Culture Collection; ATCC CRL-1573 and CRL-3216, respectively), Huh7.5.1 cells (human hepatocellular carcinoma; RRID CVCL-E049), Vero cells (monkey kidney; ATCC CRL-81), and their derivatives were cultured in DMEM with high glucose (Nacalai Tesque, Kyoto, Japan) containing 10% heat-inactivated FBS (Biowest, Bradenton, FL, USA) and 1% penicillin-streptomycin mixed solution (Nacalai Tesque). Primary HUVECs were purchased from Lonza and cultured in EBM-2 (Lonza KK, Tokyo, Japan) supplemented with EGM-2 Bulletkit (Lonza KK). All cells were maintained in 5% CO_2_ at 37°C. To establish IFNAR1-knockout HEK293 C14 cells, HEK293 cells were co-transfected with knockout plasmids pX330mEGFP-hIFNAR1-cas1 and -cas2, and EGFP-expressing cells were sorted by Aria at 4 days post-transfection. Four days after the initial sorting, ~3 × 103 U/mL IFN-alpha were added to the sorted cells and stained with mouse antibody against BST2 (BioLegend, San Diego, CA, USA) and phycoerythrin (PE)-conjugated antibody against mouse Ig after 60 h of incubation. Cells with low expression of BST2 were sorted by Aria and plated at a density of one cell per well. Each clone was treated with IFN-alpha again, and BST2 expression was analyzed using flow cytometry. Genomic DNA was also analyzed using PCR with primers hIFNAR1-F1 and -R1, showing a fragment of 131 bp in the case of complete deletion and 398 bp in wild-type cells. Finally, clone 14 was selected, showing a 267 bp deletion in the IFNAR1 gene.

### Antibodies

The antibodies were used in this study as follows: mouse monoclonal anti-human CD209 (DC-SIGN) antibody (clone 9E9A8, BioLegend), and mouse monoclonal anti-human CD299 (DC-SIGNR/L-SIGN) antibody (clone 120604, Thermo Fisher, Waltham, MA, USA) were used to detect cell surface DC-SIGN and L-SIGN, respectively. Allophycocyanin-conjugated mouse monoclonal anti-human AXL antibody (clone 108724, R&D Systems, Minneapolis, MN, USA) and goat polyclonal anti-human AXL antibody (AF154, R&D Systems) were used to detect cell surface AXL receptors. Mouse monoclonal anti-FLAG M2 antibody (F3165, Sigma Aldrich, Burlington, MA, USA) was used for detecting recombinant GAS6-FLAG protein. Goat polyclonal anti-human AXL antibody (AF154, R&D Systems) was used as a blocking antibody for SFTSV infection of HUVECs and detection of AXL by immunoblotting. Anti-SFTSV-G1 mouse monoclonal antibody (clone 51C9, Immune-Tech, New York, NY, USA) was used as a neutralizing antibody for the SFTSV infection of DC-SIGN-expressing cells. Alexa Fluor 488-conjugated donkey anti-goat antibody and Alexa Fluor 647-conjugated goat anti-mouse antibody (Invitrogen, Waltham, MA, USA) were used as secondary antibodies in flow cytometry. Horseradish peroxidase-conjugated donkey anti-goat IgG (BioLegend), -goat anti-mouse IgG (BioLegend), and -goat anti-human IgG (Jackson ImmunoResearch, West Grove, PA, USA) were used for immunoblotting.

### Plasmids, primers, and reagents

Plasmids and primers used in this study are described in Tables S2 and S3, respectively. Human TruStain FcX (BioLegend) was used to block Fc receptors. SFTSV-Gc target peptide SGc8 (SVPTGANIPSPTDWLNALFG corresponding to amino acids 450–469) was synthesized (GenScript, Piscataway, NJ, USA) (27).

### Plaque assay

Culture media (200 µL) containing 10-fold serially diluted viruses inoculated into AXL-expressing HEK293 cells in a collagen-coated 12-well plate (Iwaki Science Products Dept.) were incubated for 2 h at 37°C and washed once with culture medium. The cells were cultured in 2% FBS-DMEM containing 1% methylcellulose (Nacalai Tesque) for 7 days, fixed with 10% (vol/vol) formalin neutral-buffered solution (Nacalai Tesque), and stained with methylene blue diluted in water at room temperature (RT) for 1 h. Viral titers were expressed as plaque-forming units per milliliter.

### Preparation of pseudotype retrovirus-bearing SFTSV glycoproteins (SFTSVpv)

HEK293T cells at 80%–90% confluence in a 15 cm culture dish (Iwaki Science Products Dept.) were transfected with pCAGGS-based plasmid bearing SFTSV glycoprotein cDNA (pCAGGS-SFTSV-GPs), pCX-based plasmid bearing fluorescent reporter protein cDNA (GFP, RFP, or crimson), and retroviral packing plasmid pGP encoding gag/pol proteins (quantity ratio: pCAGGS-SFTSV-GPs:pGP:pCX-GFP/RFP/Crimson = 3:5:8) using PEI-MAX transfection reagent (Polysciences, Warrington, PA, USA). For one 15 cm dish, 11.25 µg pCAGGS-SFTSV-GPs, 18.75 µg pGP, and 30 µg pCX-GFP/RFP/crimson were diluted in 2.3 mL Opti-MEM (Thermo Fisher). The transfection reagent PEI-MAX was also diluted in 2.3 mL Opti-MEM at a final concentration of 50 mg/mL. Twelve hours after adding mixtures of plasmids and PEI-MAX, the cells were washed once and cultured in a fresh medium for 24 h. Then, the medium was collected and replaced with fresh medium with a final concentration of 20 mM HEPES (Nacalai Tesque). The next day, the medium was collected and combined with the first-time collected medium. The mixture was centrifuged at 20,400 *g* for 3 min at 4°C to remove the cell debris and stocked at −80°C in aliquots. The titration was performed as follows: HEK293 cells pre-seeded in a 12-well collagen-coated plate were incubated with 10-fold serially diluted SFTSVpv harboring fluorescent reporter genes for 12 h. After replacement with fresh medium, the cells were harvested by trypsin/ethylenediaminetetraacetic acid (EDTA) (Nacalai Tesque) at 72 hpi, and cells expressing the fluorescent protein were measured using flow cytometry, and the MOI was calculated.

### Establishment of reporter HEK293 cells bearing tetracycline-inducible dCas9-VPR (VP64, P65, and Rta)

PB-TRE-dCas9-VPR plasmid was a gift from George Church (Addgene plasmid #63800). This plasmid expresses the Tet-On 3G protein, aminoglycoside phosphotransferase for resistance to hygromycin, and Cas9m4, the inactive form of Cas9, fused with activation domains of VP64, P65, and Rta, whose expression is controlled by a third-generation tetracycline-responsive promoter that can be activated by the binding of the Tet-On 3G protein (70). Reporter HEK293 cells were stably transfected with PB-TRE-dCas9-VPR by piggyBac transposase through co-transfection with pCMV-hyPBase, a gift from the Wellcome Sanger Institute (71), in the presence of 120 µg/mL hygromycin.

### Production and titration of human genome-wide CRISPRa lentiviral library

To produce lentiviral library, the human genome-wide CRISPRa pooled libraries (a gift from Jonathan Weissman, Addgene #83978) were co-transfected with lentiviral packaging plasmids pLP-1, pLP-2, and pLP-VSVG (Thermo Fisher) into HEK293T cells (weight ratios of pLP-1:pLP-2:pLP-VSVG:CRISPRa libraries pooled plasmid = 3:1:2:4) (72). HEK293T cells were seeded and cultured until reaching 80%–90% confluence before transfection. For one 15 cm dish, a mixture of 18 µg pLP-1, 6 µg pLP-2, 12 µg pLP-VSVG, and 24 µg CRISPRa pooled libraries (14 µg Addgene #83978) was diluted in 2.3 mL Opti-MEM. PEI-MAX was diluted and incubated for 5 min at RT in 2.3 mL Opti-MEM at a final concentration of 50 mg/mL. Then, PEI-MAX and plasmid solutions were mixed and incubated for 25 min at RT. The mixture was added to the cells dropwise. After 12 h of incubation, the medium was removed, and cells were further cultured in a fresh medium. After a further 24 h, the medium was collected and replaced with fresh medium with a final concentration of 20 mM HEPES. The next day, the medium was collected and combined with the first-collected medium. The combined medium was centrifuged to remove cell debris, filtered through a membrane (Millex 0.45 µm, Millipore, Burlington, MA, USA), and stored at −80°C in aliquots. For the titration of the CRISPRa lentiviral library, ~2 × 10^5^ HEK293 cells expressing dCas9-VPR were first seeded in six-well plates (Iwaki Science Products Dept.) and cultured for 36 h before adding lentiviral supernatant. Different volumes of supernatant (50, 100, 150, 200, 300, 400, and 500 µL) were added to each well. After 12 h of incubation, the cells were further cultured in a fresh medium. After 24 h, all the cells were harvested by trypsin and split to 20% to prevent cell overgrowth. Also, the untreated control cells were simultaneously plated at different confluencies (10%, 20%, 30%, 40%, and 50%). At 72 hpi, the medium was changed to 1 µL/mL puromycin-containing medium. After a further week, the infected cells were selected by puromycin, and the MOI of the lentiviral library was calculated by comparison with the control cells.

### Infection of dCas9-VPR-expressing HEK293 cells with CRISPRa-v2 lentiviral pooled library

For large-scale screening, ~3 × 10^6^ dCas9-VPR-expressing cells were seeded in ten 15 cm dishes 48 h before infection. When the cells reached ~1 × 10^7^ cells per dish, CRISPRa-v2 lentiviral pooled library was added at an MOI of 0.3. After 12 h of incubation, the medium was replaced with fresh medium, and the cells were further cultured for 3 days. Then, the medium was supplemented with 1 µL/mL puromycin. When the cell density reached 100% confluency, the cells in the ten 15 cm dishes were combined and split to 25% confluency, and a minimum of ~1 × 10^8^ cells were seeded for culture to maintain the complexity of sgRNA libraries (coverage = 300, five sgRNA per gene for 18,915 genes). Several aliquots, including ~1 × 10^8^ cells per tube, were stocked at −80°C 2 weeks post-infection.

### Genome-wide screening of reporter cells infected with the CRISPRa-v2 lentiviral pooled library

Around 1 × 10^8^ cells (coverage = 1,000) were used for the first screening. Briefly, the cells were seeded in ten 15 cm dishes (~1 × 10^7^ cells per dish) and cultured in a medium supplemented with 1 µg/mL Dox (Sigma Aldrich). At 50% confluency (1.5–2 days after the addition of Dox), the cells were infected with SFTSVpv carrying GFP reporter gene at an MOI of 0.01 for 12 h. At 72 hpi, the cells were harvested, resuspended in 2% FBS-DMEM, and sorted in an Aria cell sorter (BD, Franklin Lakes, NJ, USA) based on GFP expression. GFP-positive cells were grown to ~1 × 10^7^ cells per dish in five 15 cm dishes. A second selection was then made in the same manner as the first, except that SFTSVpv carrying the RFP reporter gene was used instead of GFP. GFP- and RFP-positive cells were sorted and grown to ~1 × 10^7^ cells per dish in five 15 cm dishes. To confirm the performance of the screening, unsorted starting cells, first-sorted GFP-positive cells, and second-sorted GFP- and RFP-positive cells were challenged with infection by SFTSVpv carrying the crimson reporter gene at an MOI of 0.01, and 3 days after infection, the number of crimson-expressing cells was analyzed using flow cytometry. After verifying the increase in the cell number susceptible to SFTSVpv, the third selection was made in the same manner as the first and second, except that SFTSVpv carrying the crimson-reporter gene was used. After each sorting, all sorted cells were maintained in 10% FBS-DMEM containing 0.25 µg/mL puromycin.

### sgRNA library readout by deep sequencing and screening result analysis

Approximately 5 × 10^7^ unsorted control cells and 2 × 10^7^ GFP-, RFP-, and crimson-positive cells obtained by the third sorting were used for genomic DNA extraction by the Wizard Genomic DNA Purification Kit (Promega, Madison, WI, USA). Genomic DNA from control (300 µg) and sorted cells (30 µg) was used for sgRNA amplification. PCR (27 cycles) was performed using Ex Taq polymerase (Takara, Kyoto, Japan; oligos described in Table S2) and ~30 µg genomic DNA per reaction to amplify the sgRNA. PCR products in all reaction tubes were combined, and DNA bands corresponding to sgRNA were purified by applying 2% agarose gel and the Gel Extraction Kit (Qiagen, Venlo, Netherlands). After concentration of purified PCR products using a spin column (Millipore), 11.4 and 1.14 µg PCR products obtained from control and sorted cells, respectively, were mixed and analyzed by single-read next-generation sequencing (100 bp single reads and 30 M reads by NovaSeq 6000, Illumina, San Diego, CA, USA). Deep sequencing raw data were analyzed for sgRNA abundance and gene ranking by a published computational tool (73).

### Gene validation

Membrane proteins were selected for validation from the top 30 genes in the MAGeCK analysis, and these cDNAs were amplified by PCR and cloned into the retrovirus-based plasmid pMXs3-IRES2-puro. HEK293 cells were infected with a retrovirus carrying these candidate genes and selected from 72 hpi for 1 week by 1 µg/mL puromycin. The cells expressing the candidate genes were challenged with SFTSV at an MOI of 0.01 in the BSL3 facility. The total RNA was extracted from the infected cells, and the SFTSV RNA level was determined using RT-qPCR.

### RT-qPCR

Total RNA isolated from cells using the Pure-Link Mini RNA Kit (Invitrogen) was transcribed to cDNA using the ReverTra Ace qPCR RT Master Mix with gDNA Remover (Toyobo, Osaka, Japan). RT-qPCR using Thunderbird SYBR qPCR Mix (Toyobo) was performed by the QuantStudio 3 Real-Time System (Thermo Fisher). The experiment used the Comparative Ct mode, with cycling comprising 40 cycles. After an initial step at 95°C for 15 s, the temperature was then shifted to 60°C for 1 min in each cycle. The RNA expression level was normalized by HPRT1 gene mRNA, and the relative level was calculated using the ∆∆C_T_ method. The primers for RT-qPCR and raw data of RT-qPCR are listed in Table S2.

### Liposome preparation

To prepare PS-liposomes, 95 mol% 1,2-dioleoyl-sn-glycerol-3-phosphocholine (DOPC, Avanti Polar lipids, Avanti, Birmingham, AL, USA) and 5 mol% 1,2-dioleoyl-sn-glycero-3-phospho-L-serine (Avanti Polar lipids) in chloroform were dried up and then resuspended in methanol. For PC-liposomes, 100 mol% DOPC solution was dried up and resuspended in methanol. The solutions were dried up completely under a nitrogen stream, and the resulting phospholipid film in a glass vial (5 µmol) was hydrated by adding 3 mL phosphate-buffered saline (PBS), followed by vigorous vortexing for 1 h at RT. The suspensions were transferred to Eppendorf tubes and resuspended by bath sonication until the suspension was clarified; clarification occurred in 20–30 min for PS-liposomes and approximately 1 h for PC-liposomes. Finally, the liposomes were prepared by passing through the 0.4 µm polycarbonate membranes using a mini extruder system (Avanti). The extruded PC- and PS-liposomes were stocked at 4°C and used within 1 week.

### Liposome-mediated inhibition of SFTSV infection

Mock and AXL-expressing HEK293 cells were seeded in a collagen-coated 48-well plate. One day after seeding, the cells were preincubated with PC-liposomes or PS-liposomes containing 5 µmol phospholipids or PBS on ice for 15 min. After preincubation with the liposomes, the cells were infected with SFTSV at an MOI of 0.1 for 2 h at 37°C, washed three times with 2% FBS-DMEM, and cultured in fresh medium for a further 24 h. Then, total RNA was extracted and SFTSV RNA level was measured using RT-qPCR.

### Antibody-mediated inhibition of SFTSV infection

Mock, AXL-expressing, and DC-SIGN-expressing HEK293 cells were seeded in a collagen-coated 48-well plate. SFTSV was preincubated with either 0, 5, or 25 µg/mL anti-SFTSV-GPs, or control antibody in 100 µL 2% FBS-DMEM at 37°C for 1 h. The seeded cells were infected with the preincubated SFTSV at an MOI of 0.1 in the presence of these antibodies at 37°C for 2 h, washed three times with 2% FBS-DMEM, and cultured in fresh medium for a further 24 h. Then, total RNA was extracted, and SFTSV RNA level was measured using RT-qPCR. For the inhibition of AXL, HUVEC cells seeded the previous day in a collagen-coated 48-well plate were preincubated with either 0, 10, 50, or 100 nM anti-AXL antibody, or control antibody in 100 µL 2% FBS-DMEM at 37°C for 1 h, washed once, infected with SFTSV at an MOI of 0.1 at 37°C for 2 h, and cultured in fresh medium for a further 24 h. Then, total RNA was extracted, and SFTSV RNA level was measured using RT-qPCR.

### shRNA gene silencing of AXL or GAS6 expression

To achieve gene silencing, AXL-expressing HEK293 cells and HUVEC were infected with a lentivirus vector carrying shRNA to knock down GAS6 and AXL, respectively, and were selected for with blasticidin (10 µg/mL for HEK293 cells and 7.5 µg/mL for HUVEC). Knockdown efficiency was analyzed using RT-qPCR and/or flow cytometry. The plasmids used for gene knockdown are described in Table S3.

### Production of recombinant GAS6-FLAG and AXL-ΔCT-Fc proteins

To construct an expression plasmid carrying the full-length GAS6 tagged with C-terminal 3× Flag, GAS6 cDNA was amplified by PCR and cloned into the pME-3×FLAG vector. The plasmid was transfected into HEK293T cells using PEI-MAX reagent. Twelve hours after transfection, the medium was replaced with fresh 2% FBS-DMEM. After 2 days of culture, the medium was collected and used for protein purification. GAS6-FLAG was purified by anti-FLAG M2 magnetic beads (Millipore). In brief, the culture supernatant was centrifuged to remove cell debris at 180 *× g* for 5 min at 4°C, and then, the supernatant was supplemented with pre-equilibrated anti-FLAG M2 magnetic beads and incubated for at least 2 h with rotation at 4°C. The beads were washed with a washing buffer (50 mM Tris-HCl, 150 mM NaCl, pH = 7.5) three times, and the bound proteins were eluted with FLAG peptide in PBS. To obtain recombinant proteins of the AXL extracellular domain fused to the Fc portion of human IgG1 (AXL-ΔCT-Fc), regions encoding Ig1 and Ig2 domain with or without E66/T84R mutation were amplified by PCR and cloned into the pME-hIg1 plasmid. These plasmids were transfected into HEK293T cells using PEI-MAX reagent, and after 2 days of culture, the medium was collected, and AXL-ΔCT-Fc and AXL-ΔCT-E66/T84R-Fc recombinant proteins were purified using protein G magnetic beads.

### Establishment of HEK293 cells expressing wild-type and mutant AXLs

AXL cDNA with or without mutations (Y702/703A, K567M, Y821A, Y866A, Y779/821A, and AXL-∆CT) was amplified by PCR and cloned into the retrovirus-based plasmid pMXs3-IRES2-puro. The primers used for PCR are listed in Table S2. HEK293 cells infected with retrovirus carrying these AXL cDNAs were selected by 1 µg/mL puromycin for 1 week at 72 hpi and sorted by BD FACSAria cell sorter (BD) to obtain populations expressing relatively similarly high levels of AXL derivatives.

### Restoration of SFTSV infectivity of GAS6-knockdown AXL-expressing HEK293 cells by recombinant GAS6

Parental HEK293 and GAS6-knockdown AXL-expressing HEK293 cells were seeded in a collagen-coated 48-well plate and cultured in 2% FBS-DMEM for 12 h. The medium was changed to serum-free medium 1 h before infection. The cells preincubated with or without 1, 3, and 10 µg/mL GAS6 at 4°C for 1 h were infected with SFTSV at an MOI of 0.1 for 2 h in the continuous presence of GAS6 at 37°C. The total cellular RNA was extracted at 24 hpi, and relative SFTSV RNA levels were determined using RT-qPCR.

### Inhibitor treatment

The following inhibitors were used in this study: R428 (HY-15150, MCE, Monmouth Junction, NJ, USA), pictilisib (HY-50094, MCE), U73122 (HY-13419, MCE), cytochalasin D (HY-N6682, MCE), dynasore (HY-15304, MCE), CPZ (S5749, Selleck, Houston, TX, USA), pitstop 2 (HY115604, MCE), NSC23766 (HY-15723A, MCE), EIPA (L593754, MCE), and BLB (S7099, Selleck). Cells seeded in a collagen-coated 48-well plate were preincubated with inhibitors at 37°C for 1 h, infected with SFTSV at an MOI of 0.1 in the presence of inhibitors at 37°C for 2 h, washed three times with 2% FBS-DMEM, and cultured for a further 6 h in the presence of inhibitors. Then, total RNA was extracted, and SFTSV RNA level was measured using RT-qPCR.

### Immunoblotting

Cells were harvested, washed with PBS twice, and centrifuged to obtain cell pellets. The cell pellets were lysed in a lysis buffer (20 mM Tris-HCl pH = 7.5, 150 mM NaCl, 1 mM Na_2_EDTA, 1% NP-40, and 0.5% sodium deoxycholate with 1 mM phenyl methane sulfonyl fluoride) on ice for 20 min and centrifuged at 20,400 *g* at 4°C for 15 min. The supernatant of each sample was transferred to a new Eppendorf tube, mixed with 5× SDS-sample buffer, and boiled at 95°C for 5 min. Samples were applied to 7.5%–15% SDS-PAGE Gel (Nacalai Tesque). Proteins in SDS-PAGE gels were transferred to polyvinylidene difluoride (PVDF) membranes (Millipore) and treated with a blocking buffer (5% nonfat milk in Tris-buffered saline with 0.1% Tween 20; TBS-T) for 1 h. The PVDF membrane was incubated with the primary antibody (1:1,000 diluted in blocking buffer) at RT for 1 h, washed three times with TBS-T buffer, incubated with the secondary antibody (1:2,000 diluted in blocking buffer) at RT for 1 h, and washed three times with TBS-T buffer. Finally, the membrane was treated with Amersham ECL Prime Western Blotting Detection Reagent (Cytiva, Marlborough, MA, USA) and imaged by Amersham ImageQuant 800 (Cytiva). The antibodies used in immunoblotting were described in the antibodies section above.

### Flow cytometry

Cells stained with anti-AXL, anti-CD209 (DC-SIGN), and anti-CD299 (L-SIGN) antibodies (1:300 diluted) in fluorescence-activated cell sorting (FACS) buffer (PBS with 1% bovine serum albumin [BSA] and 0.1% NaN_3_) on ice for 30 min were washed twice with FACS buffer followed by staining with Alexa Fluor 488-conjugated anti-goat or Alexa Fluor 647-conjugated anti-mouse antibodies (1:600) in FACS buffer on ice for 15 min. The cells were finally washed twice with FACS buffer and analyzed by MACSQuant flow cytometer (Miltenyi Biotec, North Rhine-Westphalia, Germany) and FlowJo software (v.9.5.3, Tommy Digital, Tokyo, Japan).

### Immunofluorescence assay

To detect SFTSV in cells, mock-treated, AXL-expressing, and DC-SIGN-expressing HEK293 cells were seeded in a gelatin-coated (19895-75, Nacalai Tesque) µ-Slide 8 Well (80826, ibidi GmbH, Lochhamer Schlag, Germany). One day after seeding, the cells were infected with SFTSV at an MOI of 50 for 2 h at 37°C, washed three times with 2% FBS-DMEM, and cultured in a fresh medium for a further 24 h. Cells were fixed with 4% paraformaldehyde and permeabilized in blocking buffer (PBS with 1% BSA and 0.1% NaN_3_) with 0.1% Triton X-100 for 1 h at RT and then incubated with mouse anti-SFTSV-G1 antibody (1:250) diluted in blocking buffer with 0.1% Triton X-100 and 2.5% goat serum (PCN5000, Gibco, Thermo Fisher) for 12 h at 4°C. After washing three times in PBS, samples were incubated with Alexa 488-conjugated goat anti-mouse IgG (1:500) in blocking buffer with 0.1% Triton X-100 and 2.5% goat serum for 1 h at RT. Then, samples were incubated with Hoechst 33342 solution (19172-51, Nacalai Tesque) for 5 min at RT. After washing, coverslips were mounted in ProLong Diamond anti-fade reagent (P36982, Thermo Fisher). Images were captured using a CKX53 microscope (Olympus, Tokyo, Japan) equipped with a NY-D5600 system (Micronet Inc, Saitama, Japan).

### Quantification and statistical analysis

Statistical analysis was conducted using GraphPad Prism v.9. Data were analyzed by unpaired Student’s *t*-test, and *P*-value <0.05 was considered statistically significant (*P < 0.05; **P < 0.01; ***P < 0.001; ****P < 0.0001). Graphs were prepared in GraphPad Prism.

## Data Availability

All relevant data are available in the article and supplemental material, or upon request from the authors.

## SUPPLEMENTAL MATERIAL

Supplemental figures jvi.00221-25-s0001.docx Figures S1 and S2.

Table S1 jvi.00221-25-s0002.xlsx Raw data of genes significantly enriched in genome-wide screening.

Table S2 jvi.00221-25-s0003.xlsx Oligonucleotides used in this study.

Table S3 jvi.00221-25-s0004.xlsx Plasmids used in this study.

ASM does not own the copyrights to Supplemental Material that may be linked to, or accessed through, an article. The authors have granted ASM a non-exclusive, world-wide license to publish the Supplemental Material files. Please contact the corresponding author directly for reuse.
