# Correction for Jin et al., “SFTSV utilizes AXL/GAS6 for entry via PI3K-PLC-dependent macropinocytosis activated by AXL-kinase”

**DOI:** 10.1128/jvi.00764-26

**Published:** 2026-06-23

**Authors:** Zecheng Jin, Shuhei Taguwa, Junki Hirano, Kentaro Uemura, Chikako Ono, Akatsuki Saito, Tamaki Okabayashi, Yusuke Maeda, Taroh Kinoshita, Yoshiharu Matsuura

## AUTHOR CORRECTION

Volume 99, no. 9, e00221-25, 2025, https://doi.org/10.1128/jvi.00221-25. Acknowledgments, 2nd paragraph: “23fm0208101j0007” should read “JP25fm0208101.”

Acknowledgments, 2nd paragraph: “and the Japan Science and Technology Agency (JST) (Moonshot R&D) (JPMJMS2025)” should read “the Japan Science and Technology Agency (JST) (Moonshot R&D) (JPMJMS2025), and Takeda Science Foundation.”

