# Supplementary material for: SFTSV utilizes AXL/GAS6 for entry via PI3K-PLC-dependent macropinocytosis activated by AXL-kinase
Source: J Virol. 2025 Aug 25;99(9):e00221-25. doi: 10.1128/jvi.00221-25 (PMC12455926; doi:10.1128/jvi.00221-25)
Supplement: Supplemental figures — Figures S1 and S2. [file jvi.00221-25-s0001.docx]

**
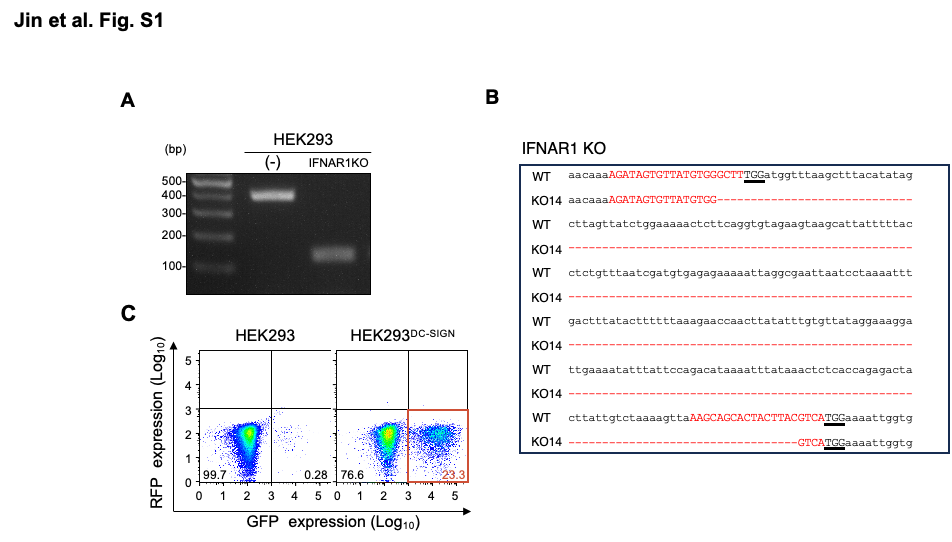
Fig. S1 Verification of IFNAR1 gene knockout and validation of the binding ability of SFTSVpv to DC-SIGN-expressing HEK293 cells**

Genomic DNA was extracted from parental HEK293 and IFNAR1-knockout HEK293 (clone 14) cells.

**(A)** The knockout target region in the IFNAR1 gene was amplified using primers hIFNAR1-F1 and hIFNAR1-R1, and the products were analyzed by agarose gel electrophoresis. The predicted sizes of the PCR products were of 398 bp and 131 bp for parental and IFNAR1-knockout HEK293 cells, respectively.

**(B)** Genomic DNA sequences of the target region in IFNAR1 gene from wild-type (WT) and IFNAR1-knockout (KO14) cells. Red letters, target sequences of IFNAR1 guide RNAs; underlined, PAM sequence.

**(C)** SFTSVpv have the binding ability to HEK293^DC-SIGN^ cells. Parental HEK293 cells and HEK293 cells expressing DC-SIGN (HEK293^DC-SIGN^) were infected with SFTSVpv harboring GFP reporter gene at an MOI of 0.01, and cells expressing GFP were assessed using flow cytometry as cell population susceptible to SFTSVpv at 72 hpi.

**
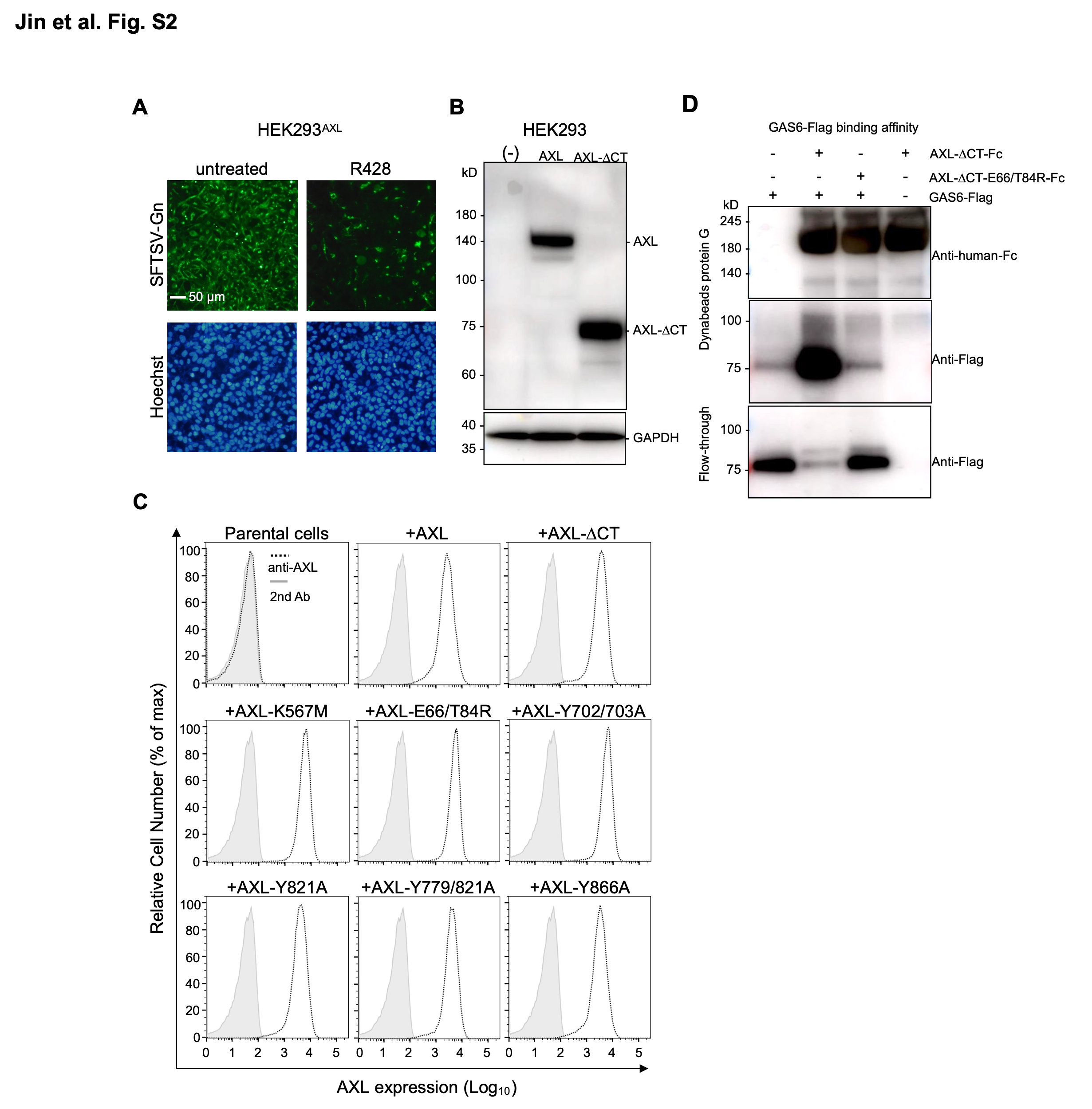
**

**Fig. S2 The intracellular domain of AXL and the binding activity to GAS6 are critical for SFTSV entry**

**(A)** Suppression of AXL-dependent SFTSV infection by AXL kinase inhibitor R428. HEK293 cells stably expressing AXL (HEK293^AXL^) were infected with SFTSV (MOI= 50) in the absence and presence of 1 mM R428 (+R428). Expression of SFTSV Gn protein (green) was determined by fluorescent microscopy at 24 hpi. Nuclei were strained by Hoechst33342 (blue). Scale bar, 50 μm.

**(B)** The HEK293 cell line stably expressing AXL lacking the C-terminal portion (ΔCT) was established, and protein expression was verified by immunoblotting.

**(C)** Similar levels of cell surface expression of various AXL mutants. HEK293 cell lines stably expressing various AXL mutants were established, and surface expression of AXL mutants was determined using flow cytometry.

**(D)** Verification of binding ability of recombinant GAS6-FLAG by immunoblotting. Recombinant Fc-fusion proteins of wild-type and E66/T84R mutant AXL extracellular domain (ECD) encoding Ig1 and Ig2 domains (AXL-ΔCT-Fc and AXL-ΔCT-E66/T84R-Fc) were pre-loaded to protein G magnetic beads, and then the beads were incubated with GAS6-Flag protein for 1 hour at 4 ℃. The binding of GAS6-Flag was assessed by immunoblotting.
